# Cost benefits of intraoperative cell salvage in radical cystectomy

**DOI:** 10.4103/0970-1591.65386

**Published:** 2010

**Authors:** Sarvpreet S. Ubee, Ramaswamy Manikandan, Adinarayana R. Gudimetla, Gurpreet Singh

**Affiliations:** Department of Urology, Southport District and General Hospital, Southport, UK

**Keywords:** Blood transfusion, intraoperative cell salvage, radical cystectomy

## Abstract

**Objective:**

We have looked into the clinical and financial benefits of using intra-operative cell salvage (ICS) as a method to reduce the amount of autologous blood transfusion (ABT) requirement for our radical cystectomy (RC) patients.

**Materials and Methods:**

Fifteen consecutive patients undergoing radical cystectomy received cell salvaged blood (ICS), while 15 did not (NCS). The cost of using the cell saver, number of homologous transfusions, survival, and recurrences were recorded and compared using paired t-test and chi-square test between the two groups. A Dideco Electa® (Sorin Group, Electa, Italy) cell saver machine was used for all the patients in the ICS group and leukocyte filters were used on the salvaged blood before the autologous transfusion.

**Results:**

The mean age was 63 years (53–72 years), 66 years (46–79 years) in ICS and NCS groups, respectively (*P* = 0.368). All 15 (100%) patients in the NCS group required an allogenic transfusion compared to 9/15 (60%) in the ICS group (*P* = 0.08). There was a significant reduction in the mean volume of allogenic blood transfused with the use of cell saver. Median follow-up was 23 and 21 months in the ICS and NCS group with 10 and 4 patients alive at last follow-up, respectively. There was a saving of 355 pounds per patient in the ICS group compared to the NCS group.

**Conclusion:**

Our initial study shows that cell savage is feasible and safe in patients undergoing radical cystectomy. It does not adversely affect the medium term outcome of patients undergoing RC and is also cost effective.

## INTRODUCTION

Radical cystectomy (RC) is the standard treatment for aggressive invasive bladder cancer. This major surgical procedure can be associated with significant intraoperative (IOP) blood loss. Age and gender, pelvic anatomy, and surgeon’s experience have an effect on the IOP blood loss.[1,2] Methods like preoperative blood donation (PBD), erythropoietin injections, acute normovolemic hemodilution (ANH), and IOP cell salvage (ICS)—all forms of autologous blood transfusion are used to compensate for blood loss and also to avoid allogenic blood transfusion (ABT). Although autologous blood abolishes the risk of alloimmunization, transfusion reactions, and transmission of infections, it has not been in favor both for clinical and practical reasons, and the drawbacks and benefits of the different modalities have been much discussed. ICS has been shown to significantly reduce the amount of ABT reactions but the theoretical risk of dissemination of cancer cells has in the past been a principal concern.[1] Studies, however, have demonstrated that ICS did not adversely affect the long-term outcomes of patients undergoing uro-oncologic surgery and there is no evidence for any cancer dissemination risk.[1,3,4] We have looked into the clinical and financial benefits of using ICS as a method to reduce the amount of ABT requirement for our RC patients.

## MATERIALS AND METHODS

A retrospective case note review of 30 consecutive patients who underwent RC was done and two groups were identified. The first group of 15 patients was operated before the purchase of cell saver and received ABT. The next 15 patients formed the ICS group, and they received cell salvaged blood. Some patients in the ICS group also received allogenic blood in the perioperative period depending upon their clinical assessment. All patients were cross-matched with allogenic blood as a part of the protocol and a standard RC or cystoprostatectomy (for male patients) with an ileal conduit was performed. A Dideco Electa^®^ (Sorin Group, Electa, Italy) cell saver machine was used for all the patients in the ICS group and leukocyte filters were used on the salvaged blood before the autologous transfusion.

Preoperative and second postoperative day hemoglobin and hematocrit levels were recorded for patients in both the groups. All patients in the ICS group received the processed autologous blood; further allogenic transfusion in the perioperative period was dependent on the clinical assessment made by the anesthetist and the surgeon, on the basis of patient's age, comorbidities, preoperative hemoglobin, and blood loss.

Postoperative follow-up was done quarterly for the first year, then 6 monthly, and then annually after 2 years. Follow-ups were done with routine blood tests and computerized tomography scans based on the agreed protocol with further management dependent on their postoperative histology and scan results. The two groups were compared with respect to their hemoglobin levels, ABT rate, and disease-free survival period using the paired *t* test and Chi-square test. We calculated the ABT costs for both the groups.

## RESULTS

The mean age in the ICS group was 63 years (53-72), as compared to 66 (46-79) in the NCS group (*P* = 0.368). The estimated mean blood loss was 2270 ml in the NCS group in comparison to 1901 ml in the ICS group (*P* = 0.3). There was no significant difference in the mean operating time (ICS 280 ± 46 min, NCS 318 ± 64; *P* = 0.1) [Table 1].

**Table 1:** Baseline characteristics of patients

	NCS	ICS
Number of patients	15	15
Age (mean)	64 years (48-79)	65 years (52-73)
Gender distribution		
Male	12	12
Female	3	3
Clinical staging		
Cis	1	3
Ta	1	1
T1	2	3
T2	8	8
T3	3	0
Hemoglobin (mean)	12.92 g/dl (9.4-14.6 g/dl)	14.43 g/dl (12-18.3 g/dl)
Hematocrit (mean)	0.389 g/dl (0.274-0.455 g/dl)	0.429 g/dl (0.359-0.541 g/dl)

All 15 (100%) patients in the NCS group required an allogenic transfusion as compared to 9/15 (60%) in the ICS group (*P* = 0.08), which was not statistically significant. Yet the mean volume of allogenic blood transfused in the ICS group was 468 ml (± 463) in contrast to 1265 ml (± 509) (*P* = 0.001) in the NCS group, and this was statistically significant. The amount of cell salvaged blood transfused was 438 ± 281 ml [Table 2]. There were two perioperative deaths in the NCS group (myocardial infarction and pulmonary embolism) and one (multiorgan failure) in the ICS group (*P* = 0.48). Median follow-up was for 23 months (5-43 months) and 21 months (4-80 months) in the ICS and NCS groups, respectively.

**Table 2:** Characteristics of the study group

	NCS	ICS	*P* value
Postoperative hemoglobin (mean)	10.72 g/dl (9.1-12.5 g/dl)	11.23 g/dl (8.7-14.8 g/dl)	
Postoperative hematocrit (mean)	0.325 g/dl (0.274-0.372 g/dl)	0.336 g/dl (0.253-0.452 g/dl)	
Postoperative staging			
Cis	1	3	
Ta	0	1	
T1	2	3	
T2	6	4	
T3	6	4	
Follow-up (months)			
Median	21	23	
Range	4-80	5-43	
Operating time (min)	318 ± 64	280 ± 46	0.1
Blood loss (ml)	2270	1901	0.3
Allogenic transfusion (no. of patients) (%)	15 (100)	9 (60)	0.08
Allogenic blood transfused (ml)	1265 ± 509	468 ± 463	0.001
Cell saver blood transfused (ml)		438 ± 281	

The machine cost was £4250, and the cost of the processing and transfusion kit for each patient in the ICS group was £77. The cost of preparation of one unit of allogenic blood is £135. When the overall costs of blood transfusion were compared between the groups, there was a saving of £355 per patient in the ICS group. The machine cost and the kit cost were recovered after the first 12 patients [Table 3].

**Table 3:** Estimation of cost for blood transfusion

	NCS	ICS
Number of allogenic blood transfusions	70	27
Estimated cost for allogenic transfusions	£9450	£3645
ICS cost for group	-	£1160
Blood transfusion cost per patient	£675	£320

## DISCUSSION

Management of IOP blood loss along with the reduction of ABT is a concern for urologists. Several authors have reported that the IOP blood loss during RC can range from 100 to 3000 ml with the allogenic transfusion rates ranging from 23% to 30%.[1,2,4,5] Nearly 50% of these patients initially start off with preoperative anemia.[2,6] While ABT rates were demonstrated to be significantly higher in men over the age of 74 years, no such difference was seen in women in a study.[5] Age did not seem to be a factor in female patients, but, overall, females had higher transfusion rates than men. Also the mean estimated blood loss (should this be estimated transfusion rate) in men was nearly 50% less[5,7] and a suggested explanation for this is that, most women have lower preoperative hemoglobin than men.

Hollenback *et al*., reviewed 2535 patients to identify the potential risk factors affecting the morbidity rates after RC; IOP blood loss and ABT were assessed to be the two major factors affecting the morbidity rates. Other factors like operative time and experience of the surgeon were identified to be modifiable risk factors. Alteration in these factors allowed for quicker patient recovery in the postoperative period.[8] The mean IOP blood loss and allogenic transfusions are related and important elements affecting the total costs of the cystectomy.[9]

ABTs carry the risk of alloimmunization and transmission of infectious diseases. The risk of transmission of hepatitis C is 1 in 30,000, and for human immunodeficiency virus (HIV) it is 1 in 200,000.[10] This risk, though low, is potentially life-threatening.

PBD became a popular alternative to allogenic transfusion when it was introduced and was assessed to be the safest option by the American Medical Association Council of Scientific Affairs.[11] This procedure is not only time consuming and time constrained but also causes inconvenience to the patient. More importantly, multicenter studies have shown a discard rate of about 50% with PBD.[12] Use of PBD also fails to avoid ABT if the IOP blood loss is more than the predicted blood loss. As up to 50% of our patients start off with anemia to consider PBD, they will require recombinant human erythropoietin, and this makes it clinically and financially unattractive.[2,12]

ANH constitutes the removal of whole blood while its volume is simultaneously replaced by either colloids or crystalloids. ANH is carried out after induction of anesthesia. The removed blood is transfused back to the patient immediately after surgery. The blood lost during surgery is hemodiluted, thus resulting in less red cells being lost. ANH has been shown in some studies to be beneficial in the avoidance allogenic transfusion.[13–15] Clinically, patients are able to tolerate ANH quite well but in some, intraoperative drop in blood pressure is a concern.[13] Preoperative anemia in patients undergoing RC also limits the use of ANH.[4,6] Takayanagi *et al*., performed RC on 97 patients and ANH was available as an option to only 42.3% of the patients due to anemia. They had an allogenic transfusion rate of 32.5%.[4]

ICS involves reinfusion of the patient's salvaged blood. The IOP blood loss is the suction recovered in the cell saver, filtered, processed, and collected in blood bags. The collected blood is transfused back to the patient either intraoperatively or in the immediate postoperative period. Its use was first described in urological surgery by Klimberg *et al*., in 1986, and patients who underwent RC were also included. Since then, the use of ICS has increased widely in urological surgeries, achieving considerable success in reducing ABT.[3,16,17]

Reinfusion of the red cells back to the patient in the perioperative period maintains an optimum hemoglobin level, which also reduces the requirement of allogenic blood in the postoperative period. Hollenbeck *et al*., demonstrated that transfusion of allogenic blood within 72 h of surgery was associated with a higher complication rate, thus increasing morbidity rates.[8]

ICS has been utilized in RC for more than two decades; however, many oncologic surgeons have been reluctant to apply it because of the theoretical risk of dissemination of cancer. Studies by various authors indicate that the survival rate of patients, who underwent RC or radical prostatectomy, is not adversely affected.[1,3,16,18] Nieder *et al*., followed 65 patients who received cell salvaged blood for a mean period of 20.7 months. The survival rate in these patients was not significantly different from the group of patients who did not receive cell salvaged blood.[1]

Stoffel *et al*., evaluated peripheral blood for prostate-specific antigen (PSA) producing cells in patients who underwent radical prostatectomy for prostate cancer, preoperatively, in the recovery room and 6 weeks postoperatively.[19] They used a reverse transcriptase polymerase chain reaction (RT-PCR) for their evaluation and found these cells in 88% of the patients in the immediate postoperative period. When these patients were tested at 6-week interval, they did not find any PSA-expressing cells and neither did they demonstrate any increased biochemical recurrence following the use of ICS. They also reported that the PSA-expressing cells are likely to suffer structural damages, thus affecting their survival.

The limitations of our study were that it was retrospective and compared a small number of patients. However, we believed that the two groups are similar as is demonstrated by the baseline characteristics. With the use of ICS, we had a reduction in the number of patients requiring allogenic transfusion, but, more importantly, the amount of ABT was significantly reduced to one-third, when compared to the NCS group. We could not eliminate the risk of ABT with ICS but we did manage to significantly reduce it. We had mortalities in both groups; patient selection with deaths in the salvage cystectomy patients was a contributory factor and this could not be attributed to the use of ICS. Results from most of the studies show that ICS is safe to use in cancer surgery; however, they are all nonrandomized studies. Only a prospective, randomized, blinded, multicenter study will be able to provide most definitive answers.

The significant reduction in the amount of ABT with the use of ICS helps to recover the cost of purchasing a cell saver machine, thereby making it cost-effective.

## CONCLUSION

The use of ICS in RC does not show any apparent increased risk of metastatic disease on medium-to long-term follow-up of these patients. It is also a very financially attractive option.
